# ﻿A new species of *Gracixalus* (Anura, Rhacophoridae) from northwestern Vietnam

**DOI:** 10.3897/zookeys.1153.93566

**Published:** 2023-03-10

**Authors:** Tung Thanh Tran, Anh Van Pham, Minh Duc Le, Nam Hai Nguyen, Thomas Ziegler, Cuong The Pham

**Affiliations:** 1 Vinh Phuc College, Phuc Yen District, Vinh Phuc Province, Vietnam; 2 Faculty of Environmental Sciences, University of Science, Vietnam National University, Hanoi, 334 Nguyen Trai Road, 11416 Hanoi, Vietnam; 3 Center for Biodiversity & Environment Research, Tay Bac University, Son La City, Son La Province, Vietnam; 4 Central Institute for Natural Resources and Environmental Studies, Vietnam National University, Hanoi,19 Le Thanh Tong, 11021 Hanoi, Vietnam; 5 Department of Herpetology, American Museum of Natural History, Central Park West at 79th Street, New York, New York 10024, USA; 6 Cologne Zoo, Riehler Straße 173, 50735, Cologne, Germany; 7 Institute of Zoology, University of Cologne, Zülpicher Straße 47b, 50674, Cologne, Germany; 8 Institute of Ecology and Biological Resources, Vietnam Academy of Science and Technology, 18 Hoang Quoc Viet Road, Hanoi, Vietnam; 9 Graduate University of Science and Technology, Vietnam Academy of Science and Technology, 18 Hoang Quoc Viet Road, Cau Giay, Hanoi, Vietnam

**Keywords:** 16S rRNA, Dien Bien Province, *Gracixalustruongi* sp. nov., morphology, Rag1, Son La Province, taxonomy

## Abstract

A new species of small tree frog is described from northwestern Vietnam based on morphological differences and molecular divergence. *Gracixalustruongi***sp. nov.** is distinguishable from its congeners and other small rhacophorid species on the basis of a combination of the following characters: size relatively small, SVL 32.2–33.1 mm in males, 37.6–39.3 mm in females; head slightly wider than long; vomerine teeth absent; snout round and long RL/SVL 0.17–0.19 in males, 0.16–0.17 in females; spines on upper eyelid absent; supratympanic fold distinct; tympanum distinct; dorsal skin smooth; throat smooth and venter granular; tibiotarsal projection absent; webbing of fingers rudimentary, toes with moderately developed webbing; dorsum moss-green, with an inverse Y-shaped dark green marking extended from interorbital region to posterior region of dorsum; external vocal sac absent in males; males with a nuptial pad on finger I. In the molecular analyses, the new species has no clear sister taxon and is at least 4.5% divergent from other congeners based on a fragment of the mitochondrial 16S rRNA gene.

## ﻿Introduction

The genus *Gracixalus* Delorme, Dubois, Grosjean & Ohler, 2005 is known from southern China through mainland Indochina, i.e., Cambodia, Laos, Vietnam, and southwards to Thailand. This genus consists of 18 recognized species, ten of which have been described in the last ten years (Frost 2022). In Vietnam, five species were recently discovered, namely *G.lumarius* Rowley, Le, Dau, Hoang & Cao, 2014; *G.sapaensis* Matsui, Ohler, Eto & Nguyen, 2017; *G.trieng* Rowley, Le, Hoang, Cao & Dau, 2020; *G.ziegleri* Le, Do, Tran, Nguyen, Orlov, Ninh & Nguyen, 2021; and *G.yunnanensis* Yu, Li, Wang, Rao, Wu & Yang, 2019. Recent phylogenetic analyses showed that there are still several unnamed distinct lineages in the *G.jinxiuensis* species group, indicating that species richness of *Gracixalus* remains underestimated (e.g., Matsui et al. 2017; Chen et al. 2018).

During our recent field work in northwestern Vietnam, specimens of a small treefrog species were collected in the karst forest of Dien Bien and Son La provinces. This treefrog taxon appears to be a member of the genus *Gracixalus* due to its small size (SVL < 40 mm), the presence of intercalary cartilage between terminal and penultimate phalanges of digits, tips of digits expanded into large discs bearing circum-marginal grooves, the vomerine teeth being absent, horizontal pupil, tibia ~ 4–5× longer than wide, translucent skin, inner (first and second) and outer (third and fourth) fingers not opposable, and dorsum with an inversed Y-shaped dark brown marking on dorsum (Fei et al. 2009; Rowley et al. 2011, 2020; Chen et al. 2018; Yu et al. 2019; Le et al. 2021). Closer examination showed that this taxon could be clearly distinguished from other known members of the genus by a combination of several morphological features in adults. In the phylogenetic analyses, this taxon forms a lineage independent from its congeners and clusters within the *Gracixalusjinxiuensis* species group with a high support level. Owing to these distinctions, we describe it herein as a new species.

## ﻿Materials and methods

### ﻿Sampling

Field surveys were conducted in September 2016 in Thuan Chau District, Son La Province; in November 2020 and December 2021 in Tuan Giao District, Dien Bien Province, northwestern Vietnam. Amphibian specimens were collected between 19:00 and 23:00 h. After having photographed the living specimens, they were anaesthetized and euthanized in a closed vessel with a piece of cotton wool containing ethyl acetate (Simmons 2002), fixed in 80% ethanol for five hours, and later transferred to 70% ethanol for permanent storage. Tissue samples were preserved separately in 70% ethanol prior to fixation. Voucher specimens referred to in this paper were deposited in the collections of the Institute of Ecology and Biological Resources (**IEBR**) and the University of Science (**HUS**), Vietnam National University (**VNU**), Hanoi, Vietnam.

### ﻿Molecular data and phylogenetic analyses

Three new samples from Dien Bien Province were included in the study. An additional 33 sequences of other species of *Gracixalus* were obtained from GenBank. Outgroup polarity was provided by three taxa, *Kurixaluseiffingeri*, *K.odontotarsus*, and *Philautusaurifasciatus* (Nguyen et al. 2013; Rowley et al. 2020). We used the protocols of Le et al. (2006) for DNA extraction, amplification, and sequencing. A fragment of the mitochondrial gene 16S was amplified using the primer pair 16Sar (5’-CGCCTGTTTATCAAAAACAT-3’) + 16Sbr (5’-CCGGTCTGAACTCAGATCACGT-3’) (Palumbi et al. 2002). To confirm genetic distinction of the new populations from other species with available samples, we also sequenced eight other species, *G.ananjevae*, *G.gracilipes*, *G.nonggangensis*, *G.quangi*, *G.sapaensis*, *G.supercornutus*, *G.trieng*, and *G.ziegleri*, using a fragment of the nuclear gene Rag1. A primer pair, Amp-RAG1 F (5’-AGCTGCAGYCARTAC CAYAARATGTA-3’) and Amp-RAG1 R1 (5’-AACTCAGCTGCATTKCCAATRTCACA-3’) (San Mauro et al. 2004), was employed to amplify DNA of targeted species. We included two species, *Philautusaurantium* and *P.ingeri*, whose sequences were obtained from GenBank, in the analyses as outgroups. After sequences were aligned by Clustal X v. 2 (Thompson et al. 1997), data were analyzed using maximum parsimony (MP) and Bayesian inference (BI), as implemented in PAUP*4.0b10 (Swofford 2001), maximum likelihood (ML), as implemented in IQ-TREE v. 1.6.7.1 (Nguyen et al. 2015), and Bayesian inference (BI), as implemented in MrBayes v. 3.2.7 (Ronquist et al. 2012) for 16S sequences. We performed BI and ML for Rag1 sequences. In addition, relationships amongst *Gracixalus* species were also inferred using the NeighborNet algorithm (Bryant and Moulton 2004) using SplitsTree v. 4.14.2 (Huson and Bryant 2006).

For MP analysis, heuristic analysis was conducted with 100 random taxon addition replicates using tree-bisection and reconnection (TBR) branch-swapping algorithm, with no upper limit set for the maximum number of trees saved. Bootstrap support was calculated using 1000 pseudo-replicates and 100 random taxon addition replicates. All characters were equally weighted and unordered. For ML analysis, we employed a single model for molecular evolution and 10,000 ultrafast bootstrap replications. The optimal model for nucleotide evolution was determined using jModeltest v. 2.1.4 (Darriba et al. 2012). For Bayesian analyses, we used the optimal model selected by jModeltest with parameters estimated by MrBayes 3.2.7. Two independent analyses with four Markov chains (one cold and three heated) were run simultaneously for 10 million generations with a random starting tree and sampled every 1000 generations. Log-likelihood scores of sample points were plotted against generation time to determine stationarity of Markov chains. Trees generated before log-likelihood scores reached stationarity were discarded from the final analyses using the burn-in function. The posterior probability values for all clades in the final majority rule consensus tree were provided. The optimal models of nucleotide evolution were set to GTR+I+G and TPM1uf+G for ML and single-modelled Bayesian analyses as selected by Modeltest v. 2.1.4 for 16S and Rag1 matrices, respectively. The cutoff points for the burn-in function was set to 73 and 32 in the Bayesian analysis, as –ln*L* scores reached stationarity after 73,000 and 32,000 generations in both runs for 16S and Rag1 datasets, respectively. Nodal support was also evaluated using bootstrap replication (BP) as estimated in PAUP, ultrafast bootstrap (UFB) in IQ-TREE, and posterior probabilities (PP) in MrBayes v. 3.2.7. BP ≥ 70 and PP and UFB ≥ 95% were regarded as strong support for a clade (Hillis and Bull 1993; Ronquist et al. 2012; Nguyen et al. 2015). Uncorrected pairwise divergences were calculated in PAUP*4.0b10.

Rag1 data of nine species were first analyzed by DnaSP v. 6.12.03 (Rozas et al. 2017) to determine sequence variation. The network analysis was then performed in SplitsTree with the following settings: edge fitting as ordinary least squares, equal angle as chosen splits transformation, least squares to modify weights and four maximum dimensions as the filtering option. The generated split graph showed a visual representation of conflicting signals in the data by presenting them as a series of parallel edges. The program computed the least squares fit (LSfit) between the pairwise distances from the graph and the distances from the matrix to produce a distance-based unrooted tree diagram by means of the neighbor-joining algorithm (Saitou and Nei 1987). The method was selected because it has been shown to outperform statistical parsimony as implemented in the software TCS (Clement et al. 2000) when the evolutionary history had many missing intermediate descents (Cassens et al. 2005).

### ﻿Morphological characters

Measurements were taken with a digital caliper to the nearest 0.1 mm. The following abbreviations were used (after Nguyen et al. 2013):

**SVL** snout-vent length;

**HL** head length (measured as a parallel line with the vertebral column from posterior margin of mandible to tip of snout);

**HW** maximum head width (at rictus);

**RL** rostral length (from anterior corner of orbit to tip of snout);

**NS** distance from nostril to the tip of snout;

**EN** distance from anterior corner of the eye to the nostril;

**IN** internarial distance;

**IOD** interorbital distance;

**ED** eye diameter;

**UEW** maximum width of upper eyelid;

**DAE** distance between anterior corner of eyes;

**DPE** distance between posterior corner of eyes;

**MFE** distance between angle of jaws and anterior corner of the eye;

**MBE** distance between angle of jaws and posterior corner of the eye;

**MN** distance from the back of mandible to the nostril;

**TYD** tympanum diameter;

**TYE** distance from anterior margin of tympanum to posterior corner of the eye;

**UAL** forelimb length (from axilla to elbow);

**FAL** hand length (from elbow to the tip of third finger);

**NPL** nuptial pad length;

**fd3** width of discs of fingers III;

**fw3** width of fingers III;

**TFL** third finger length;

**FeL** femur length (from vent to knee);

**TbL** tibia length (from knee to tarsus);

**TbW** tibia width;

**FoL** foot length (from tarsus to the tip of fourth toe);

**FTL** fourth toe length;

**IMT** inner metatarsal tubercle length;

**td4** width of discs of toes IV;

**tw4** width of toes IV.

For the webbing formula, we followed Glaw and Vences (2007). Sex was determined by gonadal inspection.

## ﻿Results

### ﻿Phylogenetic analyses

**Figure 1.**
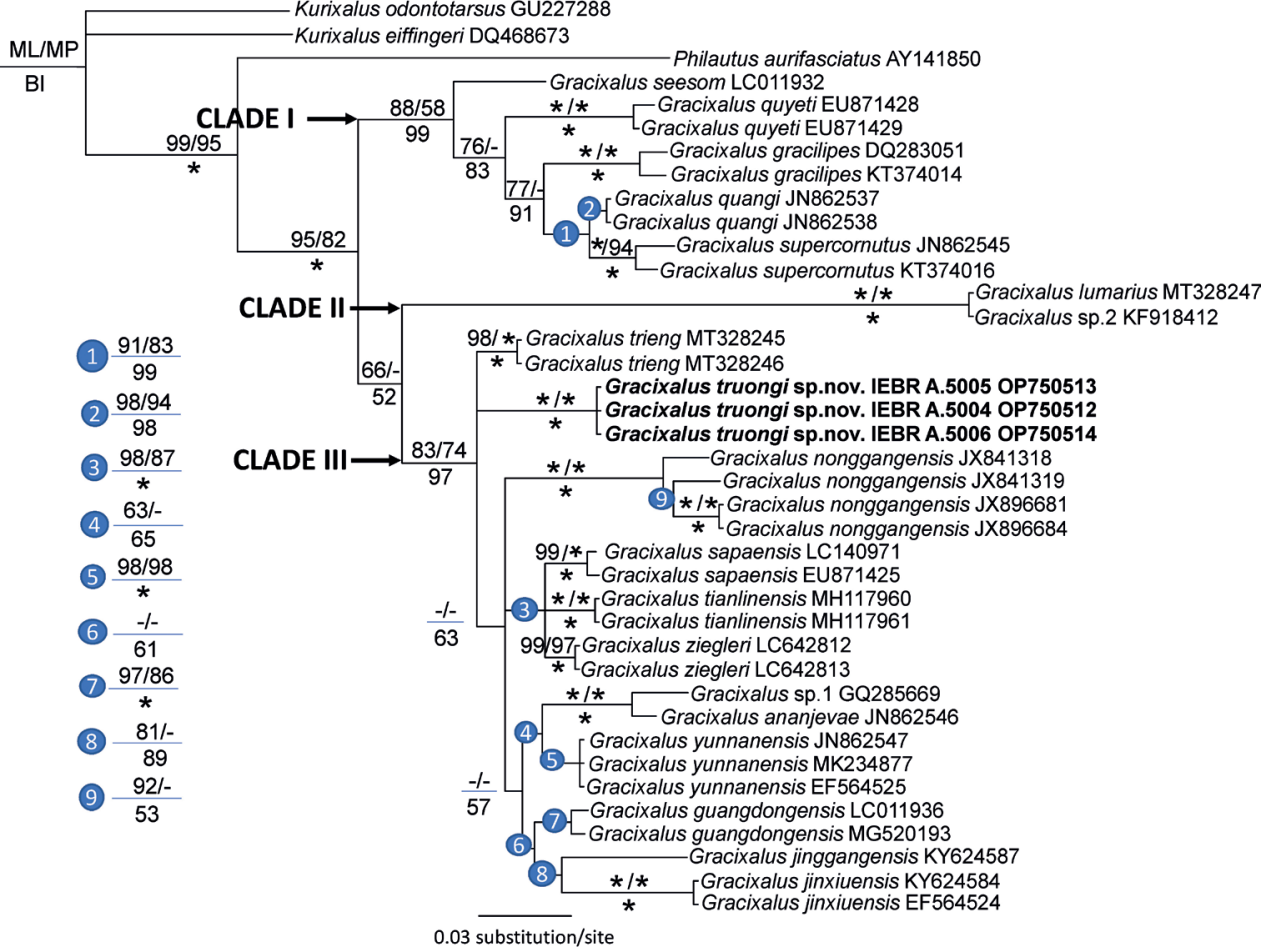
Phylogram based on the Bayesian analysis of 16S sequences. Number above and below branches are ML ultrafast bootstrap/MP bootstrap values and Bayesian posterior probabilities, respectively. Asterisk and dash represent 100% and < 50% values, respectively.

The combined matrix contained 558 aligned characters with 175 parsimony-informative sites. MP analysis of the dataset recovered the 263 most parsimonious trees with 525 steps (Consistency index = 0.51; Retention index = 0.72). Similar to Yu et al. (2019) and Le et al. (2021), our study supported the division of the genus into three distinct lineages, Clades I, II, and III, with strong nodal support from the Bayesian analysis. While Clade II was also well corroborated by ML and MP analyses, BP and UFB values for Clade I and UFB value for Clade III were insignificant. Two new populations from Dien Bien and Son La provinces were placed in Clade III along with other species from southern China and Vietnam (Fig. 1). This species is significantly divergent from others within the clade III in terms of genetic distance with the minimum pairwise divergence of approximately 4.5% based on a fragment of the mitochondrial 16S rRNA gene (Table 1 and Suppl. material 2).

**Table 1.** Uncorrected (“p”) distance matrix showing average percentage pairwise genetic divergences (%) for the 16SrRNA gene between members of the genus *Gracixalus*. The highest distance within clades is italicized and shown in parenthesis. The new species described in this paper is in bold.

		1	2	3	4	5	6	7	8	9	10	11	12	13	14	15	16	17	18	19
1	*Gracixalusananjevae*	(*0.0*)																		
2	*Gracixalusjinggangensis*	7.4	(*0.0*)																	
3	*Gracixalusjinxiuensis*	7.1	7.0	(*0.0*)																
4	*Gracixalusgracilipes*	10.6	10.8	10.7	(*1.5*)															
5	*Gracixalusguangdongensis*	4.8	5.3	5.7	10.1	(*0.7*)														
6	*Gracixaluslumarius*	14.7	14.9	16.3	15.0	14.7	(*0.0*)													
7	*Gracixalusnonggangensis*	8.2	7.5	7.8	12.3	6.7	16.5	(*2.5*)												
8	*Gracixalusquangi*	9.5	7.8	9.7	4.7	8.3	14.5	11.0	(*0.0*)											
9	*Gracixalusquyeti*	11.1	10.8	10.0	7.2	10.1	13.9	11.6	5.6	(*0.6*)										
10	*Gracixalussapaensis*	5.4	6.3	6.9	10.1	4.6	15.7	7.0	8.9	10.2	(*0.4*)									
11	*Gracixalusseesom*	10.6	9.6	9.4	6.1	8.4	16.0	10.0	5.7	7.4	8.6	(*0.0*)								
12	*Gracixalus* sp. 1	2.3	7.7	7.4	11.4	5.7	14.6	9.1	9.9	10.7	6.1	9.8	(*0.0*)							
13	*Gracixalus* sp. 2	14.0	14.9	15.9	14.3	14.7	0.0	15.7	13.9	13.8	15.3	16.1	14.6	(*0.0*)						
14	*Gracixalussupercornutus*	10.7	9.2	10.8	6.2	9.5	15.4	11.9	2.5	5.9	10.4	66	10.8	14.9	(*1.6*)					
15	*Gracixalustianlinensis*	6.3	6.5	5.9	10.5	4.5	15.2	6.8	9.6	9.6	2.7	7.6	6.7	15.3	10.6	(*0.0*)				
16	*Gracixalustrieng*	5.3	5.0	5.3	10.2	3.8	14.2	6.7	8.0	8.4	4.5	7.6	5.1	14.3	9.1	4.0	(*0.0*)			
17	*Gracixalustruongi* sp. nov.	**6.4**	**6.5**	**7.6**	**11.4**	**5.2**	**15.9**	**8.5**	**9.0**	**10.3**	**6.0**	**10.0**	**6.8**	**15.9**	**10.0**	**5.9**	**4.5**	**(*0.0*)**		
18	*Gracixalusyunnanensis*	3.9	5.0	5.8	10.1	2.2	14.3	6.6	8.0	9.7	4.3	8.7	5.1	139	8.9	4.4	4.0	**4.7**	(*0.0*)	
19	*Gracixalusziegleri*	5.4	5.7	6.1	10.5	4.5	14.9	7.1	9.5	10.3	2.3	8.2	6.1	14.9	10.5	2.5	3.7	**5.5**	4.1	(*0.0*)

**Figure 2.**
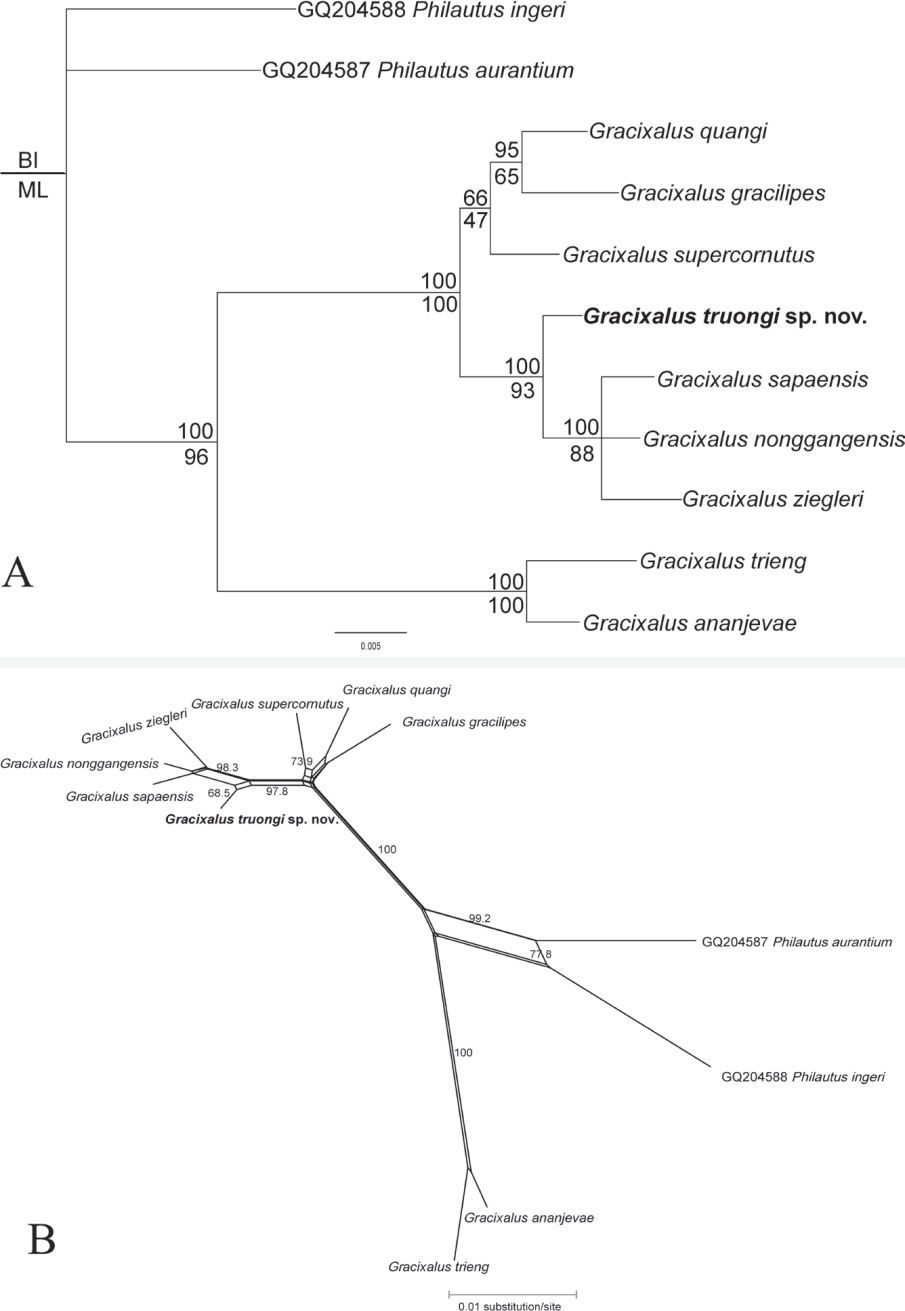
**A** phylogram based on the Bayesian analysis of Rag1 sequences. Number above and below branches are BI posterior probabilities and ML ultrafast bootstrap, respectively **B** split tree network based on Rag1 data. Numbers at major nodes are bootstrap values (1000 replicates).

The matrix of Rag1 sequences consisted of 899 aligned characters. The number of polymorphic sites was 71, parsimony-informative sites 27, and nucleotide diversity was 0.028 as determined by DnaSP. Both BI and ML analyses recovered an identical topology, although support values were generally higher in BI. The new taxon was also corroborated as a separate taxonomic unit among existing species. However, the relationships between *Gracixalus* species supported by Rag1 data differ from those in the analyses using 16S sequences. Specifically, the new taxon clustered with *G.nonggangensis*, *G.sapaensis*, and *G.ziegleri* with strong support from BI. In addition, *G.ananjevae*, and *G.trieng* formed a well-supported clade separated from the remaining congeners. The results derived from the network analysis also confirm the phylogenetic estimations (Fig. 2A, B; Suppl. materials 1, 3).

### ﻿Taxonomic account

#### ﻿ Gracixalus truongi sp. nov.

Taxon classificationAnimaliaAnuraRhacophoridae

1BC4B995-9907-59A1-8E95-44ED8015E401

https://zoobank.org/2D02EBFB-DAC0-4297-A2EC-90CF183E68FF

Figs 3 , 4

##### Material.

***Holotype***: IEBR A.5004 (Field number TN 2020.09), adult male, collected by N.H. Nguyen, H.N. Tran, H.Q. Nguyen on 11 November 2020 in the karst forest in Ta Ma Commune (21°40'36.0"N, 103°31'96.7"E, at an elevation of 1,164 m asl.), Tuan Giao District, Dien Bien Province, Vietnam. ***Paratypes***: IEBR A.5005 (Field number TN 2020.08), adult female, collected on 11 November 2020 (the same data as the holotype); IEBR A.5006 (Field number ĐB 2021.7), adult male, collected by H. Q. Nguyen & T. Q. Phan, on 30 December 2021, in Tuan Giao District, Dien Bien Province, Vietnam; ZVNU 09 (Field numbers Co9.16.24) and ZVNU 10 (Field Co9.16.36), two females, collected by A. V. Pham, N. B. Sung, L. M. Ha, T. Q. L. Hoang, and Q. T. Bui on 3 September 2016, in Long He Village (21°24'14.5"N, 103°28'41.5"E, at an elevation of 1,110 m asl.), Long He Commune, Thuan Chau District, Son La Province, Vietnam.

##### Diagnosis.

The new species is assigned to the genus *Gracixalus* based on molecular phylogenetic analyses and the following morphological characters: the presence of intercalary cartilage between terminal and penultimate phalanges of digits, tips of digits expanded into large discs bearing circum-marginal grooves, the vomerine teeth being absent, horizontal pupil, tibia ~ 4–5× longer than wide, translucent skin, inner (first and second) and outer (third and fourth) fingers not opposable, and dorsum with an inversed Y-shaped dark brown marking (Fei et al. 2009; Rowley et al. 2011, 2020; Chen et al. 2018; Yu et al. 2019; Le et al. 2021).

*Gracixalustruongi* sp. nov. is distinguishable from its congeners by a combination of the following morphological characters: (1) size relatively small, SVL 32.2–33.1 mm in males, 37.6–39.3 mm in females; (2) head slightly wider than long; (3) vomerine teeth absent; (4) snout round and long RL/SVL 0.17–0.19 in males, 0.16–0.17 in females; (5) spines on upper eyelid absent; (6) supratympanic fold distinct; (7) tympanum distinct; (8) dorsal skin smooth; (9) throat skin smooth and venter skin granular; (10) tibiotarsal projection absent; (11) webbing of fingers rudimentary, toes with moderately developed webbing; (12) dorsum moss-green, with an inverse Y-shaped dark green marking extended from interorbital region to posterior region of dorsum; (13) external vocal sac absent in males; (14) males with a nuptial pad on finger I.

**Figure 3.**
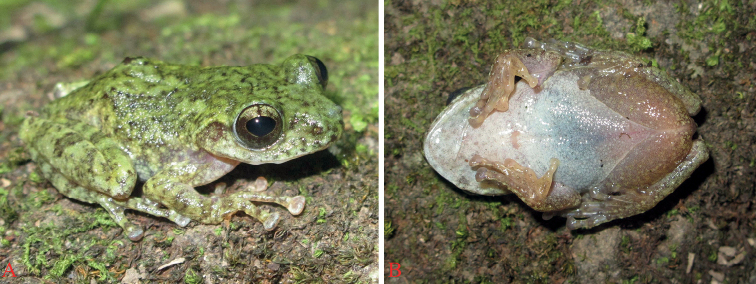
*Gracixalustruongi* sp. nov., holotype (IEBR A.5004), male, in life **A** dorsolateral view **B** ventral view.

##### Description of holotype

**(male). *Size*** small (SVL 33.1 mm), body robust, dorsoventrally compressed. Head slightly wider than long (HL 10.6 mm, HW 11.8 mm); snout round anteriorly in dorsal view, projecting beyond margin of the lower jaw; nostril round, without a lateral flap of skin, closer to tip of snout than to eye (NS 3.0 mm, EN 3.2 mm); canthus rostralis distinct and round; loreal region oblique and concave; rostral length greater than eye diameter (RL 4.8 mm, ED 4.5 mm); canthus rostralis round, loreal region oblique, concave; interorbital region flat, interorbital distance wider than internarial distance and upper eyelid width (IOD 3.9 mm, IND 3.7 mm, UEW 2.8 mm); distance between anterior corner of eyes (DAE 6.1 mm) ~ 57% distance between posterior corner of eyes (DPE 10.7 mm); pupil oval, horizontal; tympanum distinct (TYD 2.2 mm), round, half of the eye diameter but greater than tympanum-eye distance (TYE 1.5 mm); vomerine teeth absent; choanae small, oval; tongue cordate, deeply notched posteriorly; external vocal sacs absent.

***Forelimbs*** robust; forearm and hand relative long (UAL/SVL 0.16), hand longer than forearm (FAL/SVL 0.45); relative finger lengths: I<II<IV<III; fingers webbing rudimentary; dermal ridge on sides of fingers absent; tips of all fingers with well-developed discs with distinct circum-marginal grooves, discs relatively wide compared to width of finger (fd3/fw3 1.9/1.2 mm), disc of finger III smaller than tympanum diameter; subarticular tubercles markedly elevated and prominent, round, one each on fingers I and II, two on fingers III and IV; nuptial pads prominent, oval; outer palmar tubercle divided into two.

***Hindlimbs*** long (TbL/SVL 0.47, FoL/SVL 0.63); heels overlapping when held at right angles to the body; tibia length ~ 4× greater than tibia width (TbL/TbW 4.31), longer than thigh (FeL 15.1 mm) but shorter than foot length (FoL 20.8 mm); relative length of toes: I<II<III<V<IV; tips of all toes with well-developed discs with distinct circum-marginal grooves, discs slightly smaller than those of fingers; webbing formula I1–11/2II3/4–2III1–21/4IV2–1V; subarticular tubercles distinct, blunt, round: one on toes I and II, two on toes III and V, and three on toe IV; inner metatarsal tubercle small (IMT 1.3 mm); dermal ridge along outer side of tibia and tarsal fold absent; outer metatarsal and supernumerary tubercles absent; pointed projection at tibiotarsal articulation absent; tibio-tarsal articulation reaching between eye and nostril.

***Skin texture***: dorsal surface of head and body smooth; posterior part of tympanum, flank and lateral sides of limbs with small, flattened granules; spinules on upper eyelid absent; supratympanic fold distinct, extending from eye to angle of jaw; dorsolateral folds absent; throat and chest smooth, belly and ventral surface of thigh granular; dermal appendage at vent absent.

***Coloration in life***: background of dorsal surface of head, body and limbs moss-green with grey marking; with an inverse Y-shaped dark green marking, starting at the interorbital region, bifurcating into two branches on the shoulder, extending posteriorly; lateral side of body, dorsal surface of arms and limbs moss-green with dark green transverse bars; throat and chest white with dark brown marbling; belly immaculate white.

***Coloration in preservative***: Snout and dorsum grey with a dark brown pattern forming an inverse Y marking, notably a triangular pattern between eyes bifurcating into two bands continuing posteriorly; a dark pattern running from above cloaca forward to the middle of the back; lateral side of head and flank grey with dark spots; tympanum light brown; forelimb, dorsal surface of thigh, tibia and foot grey with some darker bands, posterior part of thigh below the vent yellowish brown with small white spots; throat and chest with dark brown marbling; belly immaculate cream to white; ventral part of forelimbs white; ventral surface of thighs white to grey; webbing grey.

##### Variation.

**Figure 4.**
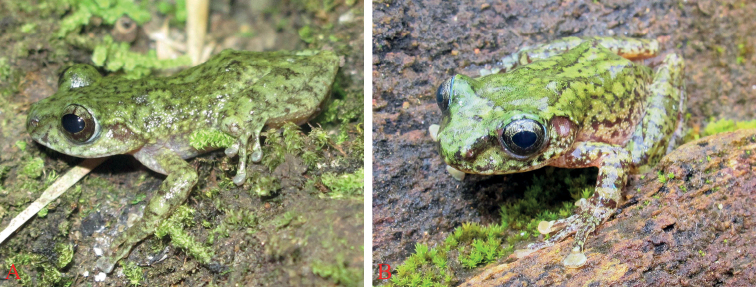
*Gracixalustruongi* sp. nov., dorsolateral view of paratypes in life **A** female (IEBR A.5005) from Dien Bien Province **B** female (ZVNU 09) from Son La Province.

Measurements and morphological characters of the type series are provided in Table 2 and photographs of the paratypes in life are presented in Fig. 4. Males are smaller than females (SVL 32.2–33.1 mm in males vs. 37.6–39.3 mm in females). The male specimens have a nuptial pad on finger I. The females contained yellowish cream eggs.

**Table 2.** Measurements (in mm) of the type series of *Gracixalustruongi* sp. nov.

	Males			Females			
	Holotype	Paratype		Paratype	Paratype	Paratype	
	IEBR A.5004	IEBR A.5006	Min – Max	IEBR A.5005	ZVNU 09	ZVNU 10	Min – Max
SVL	33.1	32.2	32.2–33.1	37.8	37.6	39.3	37.6–39.3
HW	11.8	13.0	11.8–13.0	14.6	14.6	14.8	14.6–14.8
HL	10.6	12.8	10.6–12.8	13.9	14.2	14.5	13.9–14.5
MN	9.5	10.8	9.5–10.8	11.9	12.1	12.9	11.9–12.9
MFE	7.4	8.2	7.4–8.2	9.3	9.2	9.9	9.2–9.9
MBE	4.1	5.0	4.1–5.0	4.9	5.1	5.5	4.9–5.5
RL	6.2	5.6	5.6–6.2	6.5	6.0	6.2	6.0–6.5
ED	4.5	5.1	4.5–5.1	5.1	5.1	5.3	5.1–5.3
UEW	2.8	3.2	2.8–3.2	3.7	3.5	3.9	3.5–3.9
IND	3.7	3.8	3.7–3.8	4.5	4.0	4.5	4.0–4.5
IOD	3.2	4.0	3.2–4.0	4.1	5.1	5.2	4.1–5.2
DAE	6.1	6.2	6.1–6.2	6.7	6.8	7.4	6.7–7.4
DPE	10.7	10.8	10.7–10.8	12.1	11.8	12.4	11.8–12.4
NS	3.0	2.6	2.6–3.0	3.0	2.8	2.9	2.8–3.0
EN	3.2	3.0	3.0–3.2	3.5	3.2	3.3	3.2–3.5
TYD	2.2	2.3	2.2–2.3	2.7	2.5	2.7	2.5–2.7
TYE	1.5	1.5	1.5–1.5	1.7	1.7	1.9	1.7–1.9
UAL	5.4	6.1	5.4–6.1	6.3	6.2	6.3	6.2–6.3
FAL	14.9	16.0	14.9–16.0	17.8	18.0	20.3	17.8–20.3
NPL	2.2	1.9	1.9–2.2				
TFL	8.3	8.9	8.3–8.9	8.9	8.9	10.2	8.9–10.2
fd3	1.9	1.7	1.7–1.9	2.0	1.8	1.9	1.8–2.0
fw3	1.2	1.0	1.0–1.2	1.3	1.2	1.3	1.2–1.3
FeL	15.1	15.2	15.1–15.2	17.7	17.1	20.2	17.1–20.2
TbL	15.5	18.0	15.5–18.0	18.1	19.2	21.9	18.1–21.9
TbW	3.6	4.0	3.6–4.0	4.2	4.0	4.8	4.0–4.8
FoL	20.8	23.2	20.8–23.2	24.2	25.1	28.3	24.2–28.3
FTL	12.6	13.0	12.6–13.0	14.8	15.4	16.7	14.8–16.7
td4	1.8	1.6	1.6–1.8	1.9	1.7	1.8	1.7–1.9
tw4	1.2	1.2	1.2–1.2	1.2	1.2	1.2	1.2–1.2
IMT	1.6	2.0	1.6–2.0	2.0	1.9	2.1	1.9–2.1
RL/SVL	0.19	0.17	0.17–0.19	0.17	0.16	0.16	0.16–0.17
ED/RL	0.73	0.91	0.73–0.91	0.78	0.85	0.85	0.78–0.85
TYE/TYD	0.68	0.65	0.65–0.68	0.63	0.68	0.70	0.63–0.70
UAL/SVL	0.16	0.19	0.16–0.19	0.17	0.16	0.16	0.16–0.17
FAL/SVL	0.45	0.50	0.45–0.50	0.47	0.48	0.52	0.47–0.52
TbL/TbW	4.31	4.50	4.31–4.50	4.31	4.80	4.56	4.31–4.80
TbL/SVL	0.47	0.56	0.47–0.56	0.48	0.51	0.56	0.48–0.56
FoL/SVL	0.63	0.72	0.63–0.72	0.64	0.67	0.72	0.64–0.72
fd3/TYD	0.86	0.74	0.74–0.86	0.74	0.72	0.70	0.70–0.74

##### Etymology.

We name this new species in honor of our colleague, Prof. Dr. Truong Quang Nguyen from the Institute of Ecology and Biological Resources, Vietnam Academy of Science and Technology, in recognition of his great contributions to the herpetofaunal exploration of the Indochina region. We recommend “Truong’s Treefrog” as the common English name of the new species and the common name in Vietnamese “Nhái cây trường”.

##### Ecological notes.

The specimens were collected between 19:00 and 23:00 on a limestone cliff and on leaves, ~ 0.5–1.2 m above the ground. The surrounding habitat was secondary karst forest of medium and small hardwoods mixed with shrubs and vines. Air temperature was 13–18 °C and relative humidity was 65–80%. Other amphibian species found at the site were *Leptobrachella* sp., *Kurixalusbisacculus* (Taylor, 1962), *Polypedatesmegacephalus* Hallowell, 1861, and *Rhacophorusorlovi* (Ziegler & Köhler, 2001).

##### Distribution.

**Figure 5.**
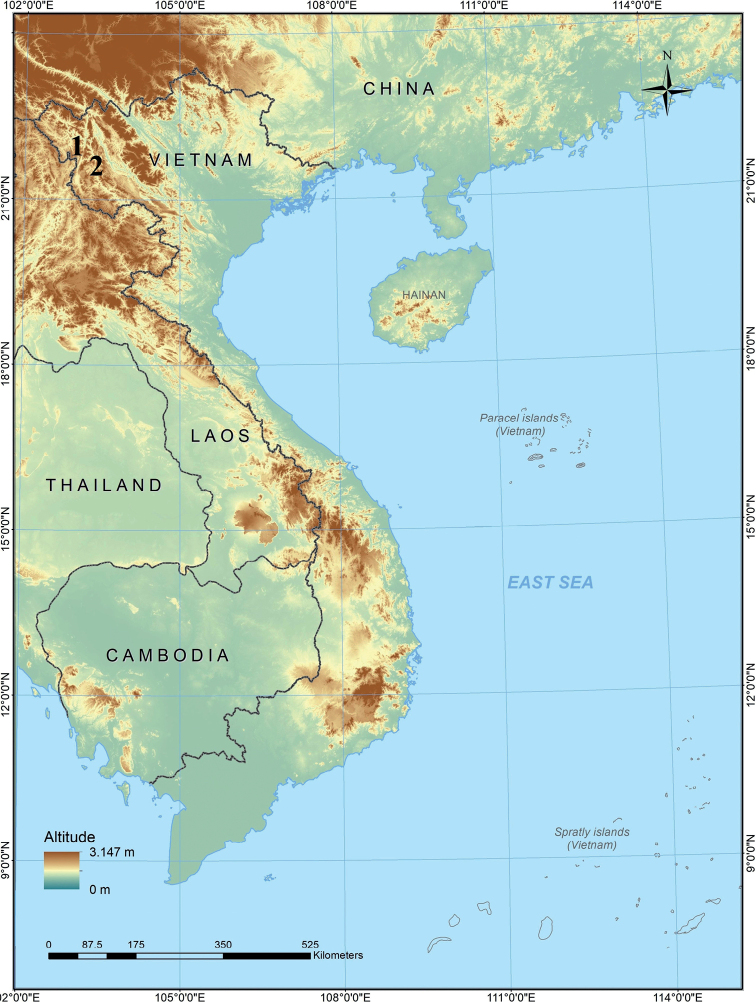
Map showing the type locality of *Gracixalustruongi* sp. nov. in Dien Bien Province (1) and the locality in Son La Province (2), Vietnam, where some of the paratypes were found.

*Gracixalustruongi* sp. nov. is currently known only from Dien Bien and Son La provinces, northwestern Vietnam (Fig. 5).

##### Comparisons.

We compared the new species with other members of the genus *Gracixalus* and data obtained from the literature (Boulenger 1893; Bourret 1937; Hu et al. 1978; Ye and Hu 1984; Matsui and Orlov 2004; Nguyen et al. 2008; Rowley et al. 2011, 2014, 2020; Mo et al. 2013; Nguyen et al. 2013; Matsui et al. 2015; Matsui et al. 2017; Zeng et al. 2017; Chen et al. 2018; Wang et al. 2018; Yu et al. 2019; Le et al. 2021) (Table 3).

**Table 3.** Morphological comparisons between *Gracixalustruongi* sp. nov., with other members of *Gracixalus.* The morphological data was obtained from the literature: Boulenger 1893; Bourret 1937; Hu et al. 1978; Ye and Hu 1984; Matsui and Orlov 2004; Nguyen et al. 2008, 2013; Rowley et al. 2011, 2014, 2020; Mo et al. 2013; Matsui et al. 2015, 2017; Zeng et al. 2017; Chen et al. 2018; Wang et al. 2018; Yu et al. 2019; Le et al. 2021). Abbreviations are as follows: ? = characters unobtainable from literature.

**Species**	**Adult male SVL (mm)**	**Adult female SVL (mm)**	**Conical tubercles on dorsum**	**Dorsal color in life**	**Vocal sac**	**Skin of body sides**	**Skin of throat**	**Finger webbing**	**Linea masculina**	**Tibiotarsal articulation**
*Gracixalustruongi* sp. nov.	32.2–33.1	37.6–39.3	absent	moss green with grey	internal	smooth	smooth	absent	absent	reaching between eye and nostril
*G.ananjevae*	20.0–32.0	43.4	absent	?	?	coarsely granular	plain	rudimentary	?	reaching eye
*G.carinensis*	30.2–38.1	?	absent	purplish, reddish, or greyish brown	internal	?	granular	rudimentary	?	reaching eye
*G.gracilipes*	20.0–24.0	26.4–28.8	absent	greenish	internal	smooth with white stripe	smooth	rudimentary	?	reaching eye
*G.guangdongensis*	26.1–34.7	34.9–35.4	absent	brown	?	rough, black blotches	granular	absent	present	reaching between eye and nostril
*G.jinggangensis*	27.9–33.8	31.6	absent	brown to beige	?	rough with tubercles	granular	rudimentary	?	reaching eye
*G.jinxiuensis*	23.5–26.3	29–30	?	brown	internal	rough with tubercles	granular	rudimentary	absent	reaching eye
*G.lumarius*	38.9–41.6	36.3	present	yellow	external	?	granular	rudimentary	?	?
*G.medogensis*	26.5	?	absent	grass green	internal	?	granular	absent	present	reaching eye
*G.nonggangensis*	27.1–35.3	26.8–27.3	absent	yellowish-olive with dark-green mark	internal	rough with tubercles	granular	absent	absent	reaching tip of snout
*G.quangi*	21.0–24.0	26.8–27.3	present, small	olive-green	external	with black blotches	smooth	absent	?	?
*G.quyeti*	28.5	34.0	present	brownish to moss-green	?	rough with sharp tubercles	smooth	rudimentary	?	reaching to snout
*G.sapaensis*	20.8–29.6	27.2–39.5	absent	Golden ochre	?	coarsely scattered with large tubercles	?	rudimentary	?	reaching eye
*G.seesom*	21.6–23.0	23.2–25.4	absent	tan	external	with large tubercles and white blotches	smooth	rudimentary	?	reaching between eye and nostril
*G.supercornutus*	22.0–24.1	?	present, bigger horn-like	green with brown spots	?	?	granular	?	?	?
*G.tianlinensis*	30.3–35.9	35.6–38.7	absent	brown to beige	external	?	granular	absent	?	?
*G.trieng*	37.2–41.4		present	brown or yellowish	present	?	granular	rudimentary	?	?
*G.yunnanensis*	26.0–34.2	?	present, small	yellow brown or red brown	external	smooth, no black blotches	granular	rudimentary	present	reaching eye
*G.ziegleri*	28.1–30.0	36.7–41.2	present	yellowish brown	internal	rough, black blotches	granular	rudimentary	absent	reaching tip of snout
**Species**	**Snout**	**White patch on temporal region**	**Tibiotarsal projection**	**Supratympanic fold**	**Venter**	**Nuptial pads**	**Heels**	**Iris**	**Linea masculina**	**Tibiotarsal articulation**
*Gracixalustruongi* sp. nov.	rounded	absent	absent	distinct	immaculate white	on finger I	overlapping	brown and moss green	absent	reaching between eye and nostril
*G.ananjevae*	slightly pointed	absent	absent	distinct	immaculate	on finger I	overlapping	?	?	reaching eye
*G.carinensis*	round	absent	absent	distinct	immaculate white	?	?	?	?	reaching eye
*G.gracilipes*	triangularly pointed	absent	absent	distinct	yellowish white	on fingers I and II	overlapping	brown	?	reaching eye
*G.guangdongensis*	triangularly pointed	present	present	distinct	throat and chest creamy white, belly light brown, semi-transparent	on finger I	overlapping	brown	present	reaching between eye and nostril
*G.jinggangensis*	triangularly pointed	absent	absent	distinct	Throat and chest dirty white with dark specks, belly white anteriorly with dark marking and posteriorly yellowish, semi-transparent	on fingers I and II	just meeting	golden	?	reaching eye
*G.jinxiuensis*	round	absent	absent	distinct	gray-brown with dark marbling	on finger I	just meeting	pale brown	absent	reaching eye
*G.lumarius*	round	absent	absent	indistinct	opaque pink	on finger I	?	dark gold	?	?
*G.medogensis*	round	absent	absent	distinct	pale green	on finger I	overlapping	?	present	reaching eye
*G.nonggangensis*	round	absent	absent	distinct	white with dark marbling, semi- transparent	on finger I	overlapping	olive	absent	reaching tip of snout
*G.quangi*	triangularly pointed	present	present	distinct	opaque white with translucent pale green margins	on finger I	?	bronze	?	?
*G.quyeti*	round	absent	absent	indistinct	belly immaculate white	?	overlapping	yellow moss green	?	reaching to snout
*G.sapaensis*	round	absent	absent	distinct	throat, chest, and belly light yellow, with dark marking	on finger I	overlapping	golden	?	reaching eye
*G.seesom*	triangularly pointed	absent	absent	distinct	anterior belly opaque white and posterior belly translucent	absent	overlapping	golden	?	reaching between eye and nostril
*G.supercornutus*	pointed	present	present	distinct	light with white spots	?	?	Pale yellow	?	?
*G.tianlinensis*	round	absent	absent	distinct	throat and chest gray with dark specks, belly creamy white, opaque	on fingers I and II	?	bronze	?	?
*G.trieng*	rounded	?	absent	distinct	throat and chest mostly yellowish brown, with dark mottling; belly pinkish brown	on fingers I and II	?	pale gold	?	?
*G.yunnanensis*	round	absent	absent	distinct	orangish with yellow spots, immaculate, semi-transparent	on finger I	overlapping	bronze	present	reaching eye
*G.ziegleri*	triangularly pointed	absent	absent	distinct	throat and chest dirty white with moderate dark specks, belly white cream with large dark blotches,	on finger I	overlapping	golden	absent	reaching tip of snout

*Gracixalustruongi* sp. nov. differs from *G.ananjevae* (Matsui & Orlov, 2004) by having skin of body sides smooth (vs. coarsely granular), snout round (vs. triangular pointed); tibio-tarsal articulation reaching between eye and nostril (vs. reaching eye). *Gracixalustruongi* sp. nov. differs from *G.carinensis* (Boulenger, 1893) by different dorsal color pattern (moss green with grey vs. purplish, reddish, or greyish brown), skin of throat smooth (vs. granular), tibio-tarsal articulation reaching between eye and nostril (vs. reaching eye). *Gracixalustruongi* sp. nov. differs from *G.gracilipes* (Bourret, 1937) by having a larger size (SVL 32.1–33.1 in males, 37.6–39.3 mm in females vs. 20.0–24.0 mm in males, 26.4–28.8 mm in females), different dorsal color pattern (moss green with grey vs. greenish with white stripe), round snout (vs. triangular pointed), tibio-tarsal articulation reaching between eye and nostril (vs. reaching eye), and iris moss green with brown marking (vs. brown). The new species differs from *G.guangdongensis* Wang, Zeng, Liu & Wang, 2018 by having a different dorsal color pattern (moss green with grey vs. brown with black blotches), skin of body sides smooth (vs. rough), skin of throat smooth (vs. granular), snout round (vs. triangular pointed), linea masculina absent (vs. present), white patch on temporal region absent (vs. present), tibiotarsal projection absent (vs. present), different venter color pattern (immaculate white vs. throat and chest creamy white, belly light brown, semi-transparent), and iris moss green with brown marking (vs. brown). *Gracixalustruongi* sp. nov. differs from *G.jinggangensis* Zeng, Zhao, Chen, Chen, Zhang & Wang, 2017 by different dorsal color pattern (moss green with grey vs. brown to beige), skin of body sides smooth (vs. rough with tubercles), skin of throat smooth (vs. granular), tibiotarsal articulation reaching between eye and nostril (vs. reaching eye), snout round (vs. triangular pointed), different venter color pattern (immaculate white vs. throat and chest dirty white with dark specks, belly white anteriorly with dark marking and posteriorly yellowish, semi-transparent), and iris moss green with brown marking (vs. golden). *Gracixalustruongi* sp. nov. differs from *G.jinxiuensis* (Hu, 1978) by having a larger size (SVL 32.1–33.1 in males, 37.6–39.3 mm in females vs. 23.5–26.3 mm in males, 29.0–30.0 mm in females), different dorsal color pattern (moss green with grey vs. brown), skin of body sides smooth (vs. rough with tubercles), skin of throat smooth (vs. granular), tibiotarsal articulation reaching between eye and nostril (vs. reaching eye), and different venter color pattern (immaculate white vs. gray-brown with dark marbling). *Gracixalustruongi* sp. nov. differs from *G.lumarius* Rowley, Le, Dau, Hoang & Cao, 2014 by having a smaller size in males (SVL 32.1–33.1 mm vs. 38.9–41.6 mm), different dorsal color pattern (moss green with grey vs. yellow), external vocal sac absent in males (vs. present), conical tubercles on dorsum absent (vs. present), skin of throat smooth (vs. granular), supratympanic fold distinct (vs. indistinct), different venter color pattern (immaculate white vs. opaque pink), and iris moss green with brown marking (vs. dark gold). *Gracixalustruongi* sp. nov. differs from *G.medogensis* (Ye & Hu, 1984) by having a larger size in males (SVL 32.1–33.1 mm vs. 26.5 mm), different dorsal color pattern (moss green with grey vs. grass green), skin of throat smooth (vs. granular), linea masculina absent (vs. present), tibio-tarsal articulation reaching between eye and nostril (vs. reaching eye), and different venter color pattern (immaculate white vs. pale green). *Gracixalustruongi* sp. nov. differs from *G.nonggangensis* Mo, Zhang, Luo, Zhou & Chen, 2013 by different dorsal color pattern (moss green with grey vs. yellowish-olive with dark-green mark), skin of body sides smooth (vs. rough with tubercles), different venter color pattern (immaculate white vs. white with dark marbling, semi-transparent), tibio-tarsal articulation reaching between eye and nostril (vs. reaching tip of snout), and iris moss green with brown marking (vs. olive). *Gracixalustruongi* sp. nov. differs from *G.quangi* Rowley, Dau, Nguyen, Cao & Nguyen, 2011 by having a larger size (SVL 32.1–33.1 in males, 37.6–39.3 mm in females vs. 21.0–24.0 mm in males, 26.8–27.3 mm in females), different dorsal color pattern (moss green with grey vs. brown with black blotches), external vocal sac absent in males (vs. present), white patch on temporal region absent (vs. present), tibiotarsal projection absent (vs. present), different venter color pattern (immaculate white vs. opaque white with translucent pale green margins), and iris moss green with brown marking (vs. bronze). *Gracixalustruongi* sp. nov. differs from *G.quyeti* (Nguyen, Hendrix, Böhme, Vu & Ziegler, 2008) by having a larger size (SVL 32.1–33.1 in males, 37.6–39.3 mm in females vs. 21.0–24.0 mm in males, 34.0 mm in the female), conical tubercles on dorsum absent (vs. present), skin of body sides smooth (vs. rough with sharp tubercles), tibio-tarsal articulation reaching between eye and nostril (vs. reaching tip of snout), and supratympanic fold distinct (vs. indistinct). The new species differs from *G.sapaensis* Matsui, Ohler, Eto & Nguyen, 2017 from by having a larger size in males (SVL 32.1–33.1 mm vs. 20.8–29.6 mm), different dorsal color pattern (moss green with grey vs. golden ochre), skin of body sides smooth (vs. coarsely scattered with large tubercles), different venter color pattern (immaculate white vs. light yellow with dark marking), tibio-tarsal articulation reaching between eye and nostril (vs. reaching eye), and iris moss green with brown marking (vs. golden). *Gracixalustruongi* sp. nov. differs from *G.seesom* Matsui, Khonsue, Panha & Eto, 2015 by having a larger size (SVL 32.1–33.1 in males, 37.6–39.3 mm in females vs. 21.6–23.0 mm in males, 23.2–25.4 mm in females), different dorsal color pattern (moss green with grey vs. tan), external vocal sacs absent in males (vs. present), round snout (vs. triangular pointed), and iris moss green with brown marking (vs. golden). *Gracixalustruongi* sp. nov. differs from *G.supercornutus* (Orlov, Ho & Nguyen, 2004) by having a larger size in males (SVL 32.1–33.1 mm vs. 22.0–24.1 mm), conical tubercles on dorsum absent (vs. present), different dorsal color pattern (moss green with grey vs. green with brown spots), skin of throat smooth (vs. granular), round snout (vs. pointed), and tibiotarsal projection absent (vs. present). *Gracixalustruongi* sp. nov. differs from *G.tianlinensis* Chen, Bei, Liao, Zhou & Mo, 2018 by having different dorsal color pattern (moss green with grey vs. brown to beige), external vocal sacs absent in males (vs. present), skin of throat smooth (vs. granular), males with a nuptial pad on finger I (vs. males with a nuptial pad on finger I and II), tibio-tarsal articulation reaching between eye and nostril (vs. reaching eye), and iris moss green with brown (vs. bronze). *Gracixalustruongi* sp. nov. differs from *G.trieng* Rowley, Le, Hoang, Cao & Dau, 2020 by having a smaller size in males (SVL 32.1–33.1 mm vs. 37.2–41.4 mm), conical tubercles on dorsum absent (vs. present), different dorsal color pattern (moss green with grey vs. yellow or yellowish), external vocal sacs absent in males (vs. present), skin of throat smooth (vs. granular), different venter color pattern (immaculate white vs. throat and chest mostly yellowish brown with dark mottling, belly pinkish brown), and iris moss green with brown (vs. pale gold). *Gracixalustruongi* sp. nov. differs from *G.yunnanensis* Yu, Li, Wang, Rao, Wu & Yang, 2019 by conical tubercles on dorsum absent (vs. present), different dorsal color pattern (moss green with grey vs. yellow brown or red brown), skin of throat smooth (vs. granular), and iris moss green with brown marking (vs. bronze). *Gracixalustruongi* sp. nov. differs from *G.ziegleri* Le, Do, Tran, Nguyen, Orlov, Ninh & Nguyen, 2021 by having a larger size in males (SVL 32.1–33.1 mm vs. 28.1–30.0 mm), conical tubercles on dorsum absent (vs. present), different dorsal color pattern (moss green with grey vs. yellowish brown with black blotches), skin of body sides smooth (vs. rough), skin of throat smooth (vs. granular), different ventral color pattern (immaculate white vs. throat and chest dirty white with moderate dark specks, belly white cream with large dark blotches), and iris moss green with brown (vs. golden).

In terms of dorsal color pattern *Gracixalustruongi* sp. nov. is similar to *Thelodermaannae* Nguyen, Pham, Nguyen, Ngo & Ziegler, 2016 from Hoa Binh anh Ninh Binh provinces. In addition, *Gracixalustruongi* sp. nov. and *Thelodermaannae* also have similar life histories, both inhabiting limestone karst forest far from water sources. However, *Gracixalustruongi* differs from *Thelodermaannae* by a larger size (SVL 32.1–33.1 in males, 37.6–39.3 mm in females vs. 27.1–28.5 mm in males, 30.3–32.6 mm in females), the presence of a dark inverse Y-marking on dorsum (vs. absent), and a higher ratio of TYD/TYD (0.67 in males and 0.67 in females vs. 0.39 in males and 0.32 in females) (Nguyen et al. 2016).

## ﻿Discussion

The discovery of *Gracixalustruongi* sp. nov. brings the number of species in the genus to a total of 19 with 13 occurring in Vietnam. It is clear that the diversity of *Gracixalus* peaks in Vietnam, including seven taxa present in the northern region, five in the central, and one in both regions of the country. The new species is most closely related to *G.trieng* in terms of genetic distance, but they are separated by 4.5% divergence based on a fragment of the mitochondrial 16S rRNA gene. Geographically, the two taxa are found in distant and distinct geographic regions. While *G.truongi* occurs in Dien Bien and Son La provinces, northwestern region, *G.trieng* is distributed in Kon Tum Province, the Central Highlands, Vietnam. In addition, the new species is recorded in the karstic landscape at elevations between 1,000 and 1,200 m, whereas *G.trieng* inhabits soil montane habitat at altitudes from 1,700 to 2,100 m. Morphologically, the former resembles *G.nonggangensis*, which occupies the same type of limestone habitat. The latter taxon is recorded between 500 and 700 m in northeastern Vietnam and 200–250 m in southern China. *G.truongi* differs from *G.nonggangensis* by 8.5% in genetic divergence based on a fragment of the mitochondrial 16S rRNA gene.

## Supplementary Material

XML Treatment for Gracixalus truongi

## Supplementary materials

Supplementary material 1 Statistical parsimony network as reconstructed by TCS v1.21 Data type figure (PDF file) https://binary.pensoft.net/file/822825This dataset is made available under the Open Database License (http://opendatacommons.org/licenses/odbl/1.0/). The Open Database License (ODbL) is a license agreement intended to allow users to freely share, modify, and use this Dataset while maintaining this same freedom for others, provided that the original source and author(s) are credited.Tung Thanh Tran, Anh Van Pham, Minh Duc Le, Nam Hai Nguyen, Thomas Ziegler, Cuong The Pham

Supplementary material 2 Uncorrected (“p”) distance matrix showing average percentage pairwise genetic divergences (%) for the 16SrRNA gene between members of the genus *Gracixalus* Data type table (word document) https://binary.pensoft.net/file/822826This dataset is made available under the Open Database License (http://opendatacommons.org/licenses/odbl/1.0/). The Open Database License (ODbL) is a license agreement intended to allow users to freely share, modify, and use this Dataset while maintaining this same freedom for others, provided that the original source and author(s) are credited.Tung Thanh Tran, Anh Van Pham, Minh Duc Le, Nam Hai Nguyen, Thomas Ziegler, Cuong The Pham

Supplementary material 3 Uncorrected (“p”) distance matrix showing average percentage pairwise genetic divergences (%) for the Rag1 gene between members of the genus *Gracixalus* Data type table (word document) https://binary.pensoft.net/file/822827This dataset is made available under the Open Database License (http://opendatacommons.org/licenses/odbl/1.0/). The Open Database License (ODbL) is a license agreement intended to allow users to freely share, modify, and use this Dataset while maintaining this same freedom for others, provided that the original source and author(s) are credited.Tung Thanh Tran, Anh Van Pham, Minh Duc Le, Nam Hai Nguyen, Thomas Ziegler, Cuong The Pham

## References

[B1] Boulenger GA (1893) Concluding report on the reptiles and batrachians obtained in Burma by Signor L. Fea dealing with the collection made in Pegu and the Karin Hills in 1887–88. Annali del Museo Civico di Storia Naturale di Genova 13: 304–347. https://doi.Org/10.5962/bhl.part.9543

[B2] Bourret R (1937) Notes herpétologiques sur l’Indochine française. XIV. Les batraciens de la collection du Laboratoire des Sciences Naturelles de l’Université. Descriptions de quinze espèces ou variétés nouvelles. Annexe au Bulletin Général de l’Instruction Publique 1937: 5–56. 10.5962/bhl.part.22065

[B3] Bryant D Moulton V (2004) Neighbor-Net: An agglomerative method for the construction of phylogenetic networks. Molecular Biology and Evolution 21(2): 255–265. 10.1093/molbev/msh018 14660700

[B4] Cassens I Mardulyn P Milinkovitch MC (2005) Evaluating intraspecific “network” construction methods using simulated sequence data: Do existing algorithms outperform the global maximum parsimony approach? Systematic Biology 54(3): 363–372. 10.1080/10635150590945377 16012104

[B5] Chen W Bei Y Liao X Zhou S Mo Y (2018) A new species of *Gracixalus* (Anura: Rhacophoridae) from West Guangxi, China. Asian Herpetological Research 9: 74–84. https://doi.Org/10.11646/zootaxa.3616.1.5

[B6] Clement M Posada D Crandall KA (2000) TCS: A computer program to estimate genealogies. Molecular Ecology 9(10): 1657–1659. 10.1046/j.1365-294x.2000.01020.x 11050560

[B7] Darriba D Taboada GL Doallo R Posada D (2012) jModelTest 2: More models, new heuristics and parallel computing. Nature Methods 9(8): e772. 10.1038/nmeth.2109 PMC4594756 22847109

[B8] Delorme M Dubois A Grosjean S Ohler A (2005) Une nouvelle classi cation générique et sub- générique de la tribu des Philautini (Amphibia, Anura, Rhacophorinae). Bulletin Mensuel de la Societe Linneenne de Lyon 74: 165–171. 10.3406/linly.2005.13595

[B9] Fei L Hu S Ye C Huang Y (2009) Fauna Sinica. Amphibia (Vol. 2). Anura, Science Press, Beijing, 887 pp.

[B10] Frost DR (2022) Amphibian Species of the World: and Online Reference. Version 6.1. American Museum of Natural History, New York. http://research.amnh.org/herpetology/amphibia/index.html/ [Accessed on 16 August 2022]

[B11] Glaw F Vences M (2007) A Field Guide to the Amphibians and Reptiles of Madagascar (3^rd^ edn.). FroschVerlag, Cologne, 496 pp.

[B12] Hillis DM Bull JJ (1993) An empirical test of bootstrapping as a method for assessing confidence in phylogenetic analysis. Systematic Biology 42(2): 182–192. 10.1093/sysbio/42.2.182

[B13] Hu S Fei L Ye C (1978) Three new amphibian species in China. Materials for Herpetological Research 4: e20.

[B14] Huson HD Bryant D (2006) Application of phylogenetic networks in evolutionary studies. Molecular Biology and Evolution 23(2): 254–267. 10.1093/molbev/msj030 16221896

[B15] Le M Raxworthy CJ McCord WP Mertz L (2006) A molecular phylogeny of tortoises (Testudines: Testudinidae) based on mitochondrial and nuclear genes. Molecular Phylogenetics and Evolution 40(2): 517–531. 10.1016/j.ympev.2006.03.003 16678445

[B16] Le DT Do YT Tran TT Nguyen TQ Orlov NL Ninh TH Nguyen TT (2021) A new species of *Gracixalus* (Anura: Rhacophoridae) from northern Vietnam. Russian Journal of Herpetology 28(3): 111–122. 10.30906/1026-2296-2021-28-3-111-122

[B17] Matsui M Orlov N (2004) A new species of *Chirixalus* from Vietnam (Anura: Rhacophoridae). Zoological Science 21(6): 671–676. 10.2108/zsj.21.671 15226589

[B18] Matsui M Khonosue W Panha S Eto K (2015) A new tree frog of the genus *Gracixalus* from Thailand (Amphibia: Rhacophoridae). Zoological Science 32(2): 204–210. 10.2108/zs140238 25826071

[B19] Matsui M Ohler A Eto K Nguyen TT (2017) Distinction of *Gracixaluscarinensis* from Vietnam and Myanmar, with description of a new species. Alytes 33: 25–37.

[B20] Mo Y Zhang W Luo Y Zhou S Chen W (2013) A new species of the genus *Gracixalus* (Amphibia: Anura: Rhacophoridae) from Southern Guangxi, China. Zootaxa 3616(1): 61–72. 10.11646/zootaxa.3616.1.5 24758792

[B21] Nguyen TQ Hendrix R Böhme W Vu TN Ziegler T (2008) A new species of the genus *Philautus* (Amphibia: Anura: Rhacophoridae) from the Truong Son Range, Quang Binh Province, central Vietnam. Zootaxa 1925(1): 1–13. 10.11646/zootaxa.1925.1.1

[B22] Nguyen TQ Le MD Pham CT Nguyen TT Bonkowski M Ziegler T (2013) A new species of *Gracixalus* (Amphibia: Anura: Rhacophoridae) from northern Vietnam. Organisms, Diversity & Evolution 13(2): 203–214. 10.1007/s13127-012-0116-0

[B23] Nguyen LT Schmidt HA von Haeseler A Bui MQ (2015) IQ-TREE: A fast and effective stochastic algorithm for estimating maximum likelihood phylogenies. Molecular Biology and Evolution 32(1): 268–274. 10.1093/molbev/msu300 25371430 PMC4271533

[B24] Nguyen TQ Pham TC Nguyen TT Ngo TT Ziegler T (2016) A new species of *Theloderma* (Amphibia: Anura: Rhacophoridae) from Vietnam. Zootaxa 4168(1): 171–186. 10.11646/zootaxa.4168.1.10 27701355

[B25] Orlov NL Ho TC Nguyen QT (2004) A new species of the genus *Philautus* from central Vietnam (Anura: Rhacophoridae). Russian Journal of Herpetology 11: 51–64.

[B26] Palumbi SR Martin A Romano S McMillan WO Stice L Grabowski G (2002) The Simple Fool’s Guide to PCR. Department of Zoology and Kewalo Marine Laboratory, Hawaii, 45 pp.

[B27] Ronquist F Teslenko M van der Mark P Ayres DL Darling A Höhna S Larget B Liu L Suchard MA Huelsenbeck JP (2012) MrBayes 3.2: Efficient Bayesian phylogenetic inference and model choice across a large model space. Systematic Biology 61(3): 539–542. 10.1093/sysbio/sys029 22357727 PMC3329765

[B28] Rowley JJL Dau QV Nguyen TT Cao TT Nguyen SN (2011) A new species of *Gracixalus* (Anura: Rhacophoridae) with a hyperextended vocal repertoire from Vietnam. Zootaxa 3125: 22–38. 10.1163/15685381-00003007

[B29] Rowley JJL Le DTT Dau VQ Hoang HD Cao TT (2014) A striking new species of phytotelm breeding tree frog (Anura: Rhacophoridae) from central Vietnam. Zootaxa 3785(1): 25–37. 10.11646/zootaxa.3785.1.2 24872168

[B30] Rowley JJL Le DTT Hoang HD Cao TT Dau QV (2020) A new species of phytotelm breeding frog (Anura: Rhacophoridae) from the Central Highlands of Vietnam. Zootaxa 4779: 341–354. 10.11646/zootaxa.4779.3.3 33055777

[B31] Rozas J Ferrer-Mata A Sánchez-DelBarrio JC Guirao-Rico S Librado P Ramos-Onsins SE Sánchez-Gracia A (2017) DnaSP 6: DNA sequence polymorphism analysis of large data sets. Molecular Biology and Evolution 34(12): 3299–3302. 10.1093/molbev/msx248 29029172

[B32] Saitou N Nei M (1987) The neighbour-joining method: A new method for reconstructing phylogenetic trees. Molecular Biology and Evolution 4: 406–425. 3447015 10.1093/oxfordjournals.molbev.a040454

[B33] San Mauro D Gower DJ Oommen OV Wilkinson M Zardoya R (2004) Phylogeny of caecilian amphibians (Gymnophiona) based on complete mitochondrial genomes and nuclear RAG1. Molecular Phylogenetics and Evolution 33(2): 413–427. 10.1016/j.ympev.2004.05.014 15336675

[B34] Simmons JE (2002) Herpetological collecting and collections management. Revised edition. Society for the Study of Amphibians and Reptiles. Herpetological Circular 31: 1–153.

[B35] Swofford DL (2001) PAUP: Phylogenetic Analysis Using Parsimony (*and other Methods), Version 4.0. Sinauer Associates, Sunderland.

[B36] Thompson JD Gibson TJ Plewniak F Jeanmougin F Higgins DG (1997) The ClustalX windows interface: Xexible strategies for multiple sequence alignment aided by quality analysis tools. Nucleic Acids Research 25(24): 4876–4882. 10.1093/nar/25.24.4876 9396791 PMC147148

[B37] Wang J Zeng Z Lyu Z Liu Z Wang Y (2018) Description of a new species of *Gracixalus* (Amphibia: Anura: Rhacophoridae) from Guangdong Province, southeastern China. Zootaxa 4420(2): 251–269. 10.11646/zootaxa.4420.2.7 30313546

[B38] Ye C Hu S (1984) A new species of *Philautus* (Anura: Rhacophoridae) from Xizang Autonomous Region. Acta Herpetologica Sinica 3: 67–69.

[B39] Yu G Hui H Wang J Rao D Wu Z Yang J (2019) A new species of *Gracixalus* (Anura, Rhacophoridae) from Yunnan, China. ZooKeys 851: 91–111. 10.3897/zookeys.851.32157 31205444 PMC6557903

[B40] Zeng Z Zhao J Chen C Chen G Zhang Z Wang Y (2017) A new species of the genus *Gracixalus* (Amphibia: Anura: Rhacophoridae) from Mount Jinggang, southeastern China. Zootaxa 4250(2): 171–185. 10.11646/zootaxa.4250.2.3 28610025

