# Supplementary figures and images for: ﻿A new species of Gracixalus (Anura, Rhacophoridae) from northwestern Vietnam
Source: Zookeys. 2023 Mar 10;1153:15–35. doi: 10.3897/zookeys.1153.93566 (PMC10208806; doi:10.3897/zookeys.1153.93566)

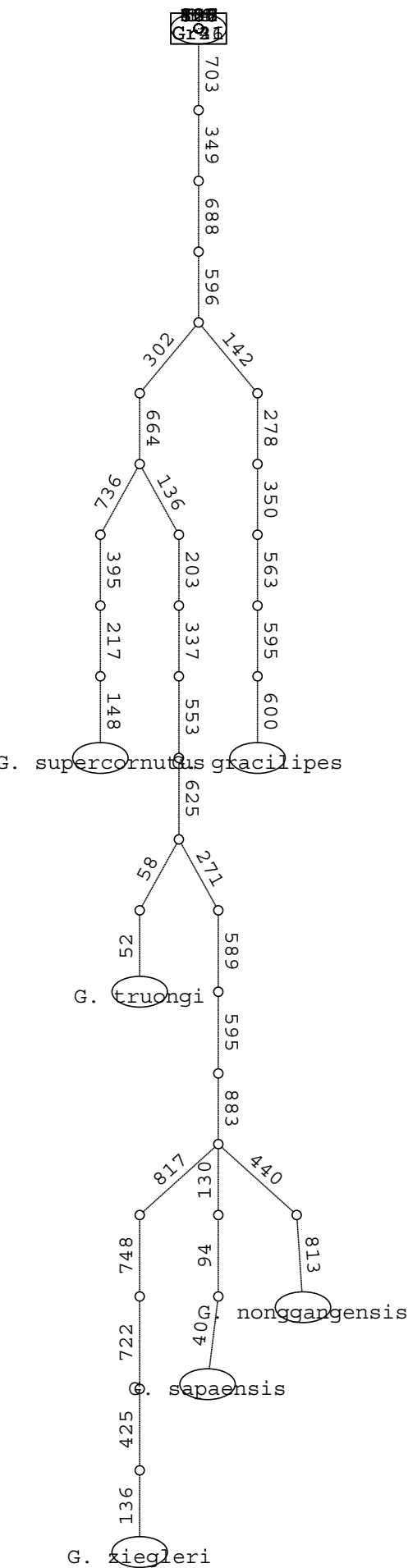

Supplement: Supplementary material 1 — Statistical parsimony network as reconstructed by TCS v1.21 [file zookeys-1153-015_article-93566__-s001.pdf]
