# Supplementary material for: ﻿A new species of Gracixalus (Anura, Rhacophoridae) from northwestern Vietnam
Source: Zookeys. 2023 Mar 10;1153:15–35. doi: 10.3897/zookeys.1153.93566 (PMC10208806; doi:10.3897/zookeys.1153.93566)
Supplement: Supplementary material 2 — Uncorrected (“p”) distance matrix showing average percentage pairwise genetic divergences (%) for the 16SrRNA gene between members of the genus Gracixalus [file zookeys-1153-015_article-93566__-s002.docx]

**Supplementary Table S1:** Uncorrected (“p”) distance matrix showing average percentage pairwise genetic divergences (%) for the 16SrRNA gene between members of the genus *Gracixalus*.

|  |  |  | 1 | 2 | 3 | 4 | 5 | 6 | 7 | 8 | 9 | 10 | 11 | 12 | 13 | 14 | 15 | 16 | 17 | 18 |
| --- | --- | --- | --- | --- | --- | --- | --- | --- | --- | --- | --- | --- | --- | --- | --- | --- | --- | --- | --- | --- |
| 1 | *Gracixalus ananjevae* JN862546 |  | - |  |  |  |  |  |  |  |  |  |  |  |  |  |  |  |  |  |
| 2 | *G. jinggangensis KY624587* |  | 7.365 | - |  |  |  |  |  |  |  |  |  |  |  |  |  |  |  |  |
| 3 | *G. jinxiuensis* EF564524 |  | 7.10 | 6.96 | - |  |  |  |  |  |  |  |  |  |  |  |  |  |  |  |
| 4 | *G. jinxiuensis* KY624584 |  | 7.15 | 6.88 | 0.00 | - |  |  |  |  |  |  |  |  |  |  |  |  |  |  |
| 5 | *G. gracilipes* DQ283051 |  | 10.45 | 11.03 | 10.62 | 10.77 | - |  |  |  |  |  |  |  |  |  |  |  |  |  |
| 6 | *G. gracilipes* KT374014 |  | 10.64 | 10.59 | 10.80 | 10.77 | 1.46 | - |  |  |  |  |  |  |  |  |  |  |  |  |
| 7 | *G. guangdongensis* MG520193 |  | 5.00 | 5.22 | 5.63 | 5.63 | 10.14 | 9.92 | - |  |  |  |  |  |  |  |  |  |  |  |
| 8 | *G. guangdongensis* LC011936 |  | 4.58 | 5.29 | 5.80 | 5.76 | 10.50 | 10.01 | 0.73 | - |  |  |  |  |  |  |  |  |  |  |
| 9 | *G. lumarius* MT328247 |  | 14.67 | 14.86 | 15.98 | 16.50 | 15.45 | 14.50 | 14.62 | 14.70 | - |  |  |  |  |  |  |  |  |  |
| 10 | *G. nonggangensis* JX841318 |  | 8.58 | 7.60 | 7.66 | 7.53 | 11.59 | 11.40 | 6.89 | 7.69 | 16.08 | - |  |  |  |  |  |  |  |  |
| 11 | *G. nonggangensis* JX841319 |  | 7.63 | 7.15 | 8.53 | 8.17 | 12.45 | 12.26 | 5.63 | 6.25 | 16.57 | 2.54 | - |  |  |  |  |  |  |  |
| 12 | *G. nonggangensis* JX896681 |  | 8.37 | 7.57 | 7.82 | 7.54 | 13.00 | 12.27 | 6.47 | 6.98 | 16.60 | 2.37 | 2.53 | - |  |  |  |  |  |  |
| 13 | *G. nonggangensis* JX896684 |  | 8.37 | 7.57 | 7.82 | 7.54 | 13.00 | 12.27 | 6.47 | 6.98 | 16.60 | 2.37 | 2.53 | 0.00 | - |  |  |  |  |  |
| 14 | *G. quangi* JN862537 |  | 9.54 | 7.83 | 9.70 | 9.72 | 4.74 | 4.74 | 8.22 | 8.27 | 14.53 | 10.30 | 10.80 | 11.36 | 11.36 | - |  |  |  |  |
| 15 | *G. quangi* JN862538 |  | 9.54 | 7.83 | 9.70 | 9.72 | 4.74 | 4.74 | 8.22 | 8.27 | 14.53 | 10.30 | 10.80 | 11.36 | 11.36 | 0.00 | - |  |  |  |
| 16 | *G. quyeti* EU871428 |  | 10.80 | 11.16 | 9.62 | 10.09 | 7.47 | 7.25 | 10.30 | 9.94 | 14.16 | 11.23 | 11.77 | 11.96 | 11.96 | 5.90 | 5.90 | - |  |  |
| 17 | *G. quyeti* EU871429 |  | 11.40 | 10.53 | 9.82 | 10.30 | 7.27 | 6.65 | 10.09 | 10.18 | 13.57 | 11.03 | 11.57 | 11.76 | 11.76 | 5.30 | 5.30 | 0.59 | - |  |
| 18 | *G. sapaensis* EU871425 |  | 5.45 | 6.25 | 6.41 | 6.66 | 10.17 | 9.95 | 4.58 | 4.57 | 15.03 | 7.02 | 6.80 | 7.00 | 7.00 | 8.79 | 8.79 | 9.93 | 9.35 | - |
| 19 | *G. sapaensis* LC140971 |  | 5.32 | 6.27 | 7.10 | 7.44 | 10.46 | 10.00 | 4.91 | 4.49 | 16.37 | 7.67 | 6.61 | 6.81 | 6.81 | 8.87 | 8.87 | 11.10 | 10.43 | 0.44 |
| 20 | *G. seesom* LC011932 |  | 10.62 | 9.60 | 9.44 | 9.42 | 6.24 | 6.00 | 8.26 | 8.55 | 16.01 | 9.90 | 10.12 | 10.09 | 10.09 | 5.74 | 5.74 | 7.79 | 7.11 | 8.53 |
| 21 | *G*. sp.1 GQ285669 |  | 2.26 | 7.74 | 7.55 | 7.28 | 11.44 | 11.44 | 5.81 | 5.52 | 14.63 | 8.93 | 8.50 | 9.48 | 9.48 | 9.92 | 9.92 | 10.40 | 10.99 | 5.80 |
| 22 | *G.* sp.2 KF918412 |  | 14.04 | 14.89 | 15.29 | 16.54 | 14.79 | 13.88 | 14.65 | 14.73 | 0.00 | 15.36 | 15.84 | 15.87 | 15.87 | 13.88 | 13.88 | 14.05 | 13.46 | 14.95 |
| 23 | *G. supercornutus* JN862545 |  | 10.80 | 9.26 | 10.78 | 10.95 | 6.39 | 6.39 | 9.24 | 9.45 | 15.64 | 11.39 | 11.89 | 12.44 | 12.44 | 2.74 | 2.74 | 6.09 | 5.89 | 10.33 |
| 24 | *G. supercornutus* KT374016 |  | 10.62 | 9.09 | 10.60 | 10.77 | 6.02 | 6.02 | 9.48 | 9.72 | 15.07 | 10.84 | 11.34 | 12.26 | 12.26 | 2.19 | 2.19 | 6.11 | 5.51 | 9.95 |
| 25 | *G. tianlinensis* MH117960 |  | 6.32 | 6.52 | 5.90 | 5.85 | 10.73 | 10.34 | 4.18 | 4.83 | 15.23 | 7.09 | 7.05 | 6.49 | 6.49 | 9.57 | 9.57 | 9.89 | 9.29 | 2.56 |
| 26 | *G. tianlinensis* MH117961 |  | 6.32 | 6.51 | 5.90 | 5.85 | 10.72 | 10.34 | 4.17 | 4.82 | 15.21 | 7.08 | 7.04 | 6.49 | 6.49 | 9.57 | 9.57 | 9.89 | 9.28 | 2.56 |
| 27 | *G. trieng* MT328245 |  | 5.34 | 5.03 | 5.52 | 5.22 | 10.34 | 9.95 | 3.95 | 3.62 | 14.24 | 6.89 | 6.66 | 6.68 | 6.68 | 8.05 | 8.05 | 8.27 | 8.47 | 4.28 |
| 28 | *G. trieng* MT328246 |  | 5.34 | 5.03 | 5.52 | 5.22 | 10.34 | 9.95 | 3.95 | 3.62 | 14.24 | 6.89 | 6.66 | 6.68 | 6.68 | 8.05 | 8.05 | 8.27 | 8.47 | 4.28 |
| **29** | ***Gracixalus* sp. nov. IEBRA5004** |  | **6.42** | **6.47** | **7.72** | **7.49** | **11.60** | **11.21** | **5.19** | **5.25** | **15.95** | **8.52** | **8.47** | **8.50** | **8.50** | **8.94** | **8.94** | **10.22** | **10.41** | **5.61** |
| **30** | ***Gracixalus* sp. nov. IEBRA5005** |  | **6.43** | **6.47** | **7.74** | **7.48** | **11.62** | **11.24** | **5.18** | **5.24** | **15.95** | **8.54** | **8.50** | **8.52** | **8.52** | **8.97** | **8.97** | **10.24** | **10.44** | **5.62** |
| **31** | ***Gracixalus* sp. nov. IEBRA5006** |  | **6.43** | **6.47** | **7.74** | **7.48** | **11.62** | **11.24** | **5.18** | **5.24** | **15.95** | **8.54** | **8.50** | **8.52** | **8.52** | **8.97** | **8.97** | **10.24** | **10.44** | **5.62** |
| 32 | *G. yunnanensis* EF564525 |  | 3.82 | 5.04 | 5.63 | 5.87 | 10.25 | 9.88 | 2.27 | 2.15 | 14.27 | 6.93 | 6.35 | 6.36 | 6.36 | 8.05 | 8.05 | 9.63 | 9.83 | 4.46 |
| 33 | *G. yunnanensis* JN862547 |  | 3.82 | 5.04 | 5.63 | 5.87 | 10.25 | 9.88 | 2.27 | 2.15 | 14.27 | 6.93 | 6.35 | 6.36 | 6.36 | 8.05 | 8.05 | 9.63 | 9.83 | 4.46 |
| 34 | *G. yunnanensis* MK234877 |  | 3.99 | 5.03 | 5.88 | 5.85 | 10.71 | 10.33 | 2.28 | 2.16 | 14.25 | 7.25 | 6.65 | 6.67 | 6.67 | 8.42 | 8.42 | 9.69 | 9.89 | 4.47 |
| 35 | *G. ziegleri* LC642812 |  | 5.33 | 5.61 | 6.13 | 6.08 | 10.66 | 10.25 | 4.25 | 4.69 | 14.84 | 7.37 | 6.96 | 6.95 | 6.95 | 9.42 | 9.42 | 10.57 | 9.92 | 2.33 |
| 36 | *G. ziegleri* LC642813 |  | 5.38 | 5.67 | 6.19 | 6.15 | 10.77 | 10.36 | 4.29 | 4.75 | 14.99 | 7.45 | 7.03 | 7.02 | 7.02 | 9.51 | 9.51 | 10.68 | 10.03 | 2.35 |

|  |  | 19 | 20 | 21 | 22 | 23 | 24 | 25 | 26 | 27 | 28 | **29** | **30** | **31** | 32 | 33 | 34 | 35 | 36 |
| --- | --- | --- | --- | --- | --- | --- | --- | --- | --- | --- | --- | --- | --- | --- | --- | --- | --- | --- | --- |
| 19 | *G. sapaensis* LC140971 | - |  |  |  |  |  |  |  |  |  |  |  |  |  |  |  |  |  |
| 20 | *G. seesom* LC011932 | 8.74 | - |  |  |  |  |  |  |  |  |  |  |  |  |  |  |  |  |
| 21 | *G*. sp.1 GQ285669 | 6.42 | 9.83 | - |  |  |  |  |  |  |  |  |  |  |  |  |  |  |  |
| 22 | *G.* sp.2 KF918412 | 15.58 | 16.06 | 14.60 | - |  |  |  |  |  |  |  |  |  |  |  |  |  |  |
| 23 | *G. supercornutus* JN862545 | 10.82 | 6.87 | 10.66 | 15.14 | - |  |  |  |  |  |  |  |  |  |  |  |  |  |
| 24 | *G. supercornutus* KT374016 | 10.39 | 6.42 | 10.86 | 14.60 | 1.64 | - |  |  |  |  |  |  |  |  |  |  |  |  |
| 25 | *G. tianlinensis* MH117960 | 2.91 | 7.57 | 6.65 | 15.27 | 10.91 | 10.35 | - |  |  |  |  |  |  |  |  |  |  |  |
| 26 | *G. tianlinensis* MH117961 | 2.91 | 7.57 | 6.65 | 15.26 | 10.90 | 10.34 | 0.00 | - |  |  |  |  |  |  |  |  |  |  |
| 27 | *G. trieng* MT328245 | 4.73 | 7.58 | 5.12 | 14.29 | 9.17 | 8.99 | 3.98 | 3.98 | - |  |  |  |  |  |  |  |  |  |
| 28 | *G. trieng* MT328246 | 4.73 | 7.58 | 5.12 | 14.29 | 9.17 | 8.99 | 3.98 | 3.98 | 0.00 | - |  |  |  |  |  |  |  |  |
| **29** | ***Gracixalus* sp. nov. IEBRA5004** | **6.37** | **10.04** | **6.79** | **15.88** | **9.70** | **10.27** | **5.90** | **5.89** | **4.55** | **4.55** | **-** |  |  |  |  |  |  |  |
| **30** | ***Gracixalus* sp. nov. IEBRA5005** | **6.38** | **10.03** | **6.79** | **15.92** | **9.73** | **10.30** | **5.89** | **5.88** | **4.54** | **4.54** | **0.00** | **-** |  |  |  |  |  |  |
| **31** | ***Gracixalus* sp. nov. IEBRA5006** | **6.38** | **10.03** | **6.79** | **15.92** | **9.73** | **10.30** | **5.89** | **5.88** | **4.54** | **4.54** | **0.00** | **0.00** | **-** |  |  |  |  |  |
| 32 | *G. yunnanensis* EF564525 | 4.03 | 8.75 | 5.09 | 13.65 | 8.95 | 8.77 | 4.40 | 4.39 | 3.99 | 3.99 | **4.71** | **4.71** | **4.71** | - |  |  |  |  |
| 33 | *G. yunnanensis* JN862547 | 4.03 | 8.75 | 5.09 | 13.65 | 8.95 | 8.77 | 4.40 | 4.39 | 3.99 | 3.99 | **4.71** | **4.71** | **4.71** | 0.00 | - |  |  |  |
| 34 | *G. yunnanensis* MK234877 | 4.23 | 8.73 | 5.12 | 14.28 | 9.16 | 8.99 | 4.37 | 4.36 | 3.98 | 3.98 | **4.73** | **4.73** | **4.73** | 0.00 | 0.00 | - |  |  |
| 35 | *G. ziegleri* LC642812 | 2.23 | 8.23 | 6.10 | 14.88 | 10.62 | 10.21 | 2.44 | 2.44 | 3.68 | 3.68 | **5.48** | **5.47** | **5.47** | 4.09 | 4.09 | 4.05 | - |  |
| 36 | *G. ziegleri* LC642813 | 2.26 | 8.33 | 6.15 | 15.02 | 10.72 | 10.31 | 2.47 | 2.47 | 3.71 | 3.71 | **5.54** | **5.52** | **5.52** | 4.13 | 4.13 | 4.09 | 0.00 | - |
