# Supplementary material for: ﻿A new species of Gracixalus (Anura, Rhacophoridae) from northwestern Vietnam
Source: Zookeys. 2023 Mar 10;1153:15–35. doi: 10.3897/zookeys.1153.93566 (PMC10208806; doi:10.3897/zookeys.1153.93566)
Supplement: Supplementary material 3 — Uncorrected (“p”) distance matrix showing average percentage pairwise genetic divergences (%) for the Rag1 gene between members of the genus Gracixalus [file zookeys-1153-015_article-93566__-s003.doc]

**Supplementary Table S2**: Uncorrected (“p”) distance matrix showing average percentage pairwise genetic divergences (%) for the Rag1 gene between members of the genus *Gracixalus*.

|  |  | 1 | 2 | 3 | **4** | 5 | 6 | 7 | 8 | 9 | 10 | 11 |
| --- | --- | --- | --- | --- | --- | --- | --- | --- | --- | --- | --- | --- |
| 1 | GQ204587 *Philautus aurantium* | - |  |  |  |  |  |  |  |  |  |  |
| 2 | GQ204588 *Philautus ingeri* | 3.01 | - |  |  |  |  |  |  |  |  |  |
| 3 | *Gracixalus quangi* | 4.35 | 4.71 | - |  |  |  |  |  |  |  |  |
| **4** | ***Gracixalus truongi* sp. nov.** | **4.27** | **4.78** | **1.51** | **-** |  |  |  |  |  |  |  |
| 5 | *Gracixalus supercornutus* | 4.21 | 4.69 | 1.12 | **1.27** | - |  |  |  |  |  |  |
| 6 | *Gracixalus trieng* | 5.04 | 5.16 | 4.90 | **5.10** | 4.89 | - |  |  |  |  |  |
| 7 | *Gracixalus gracilipes* | 4.32 | 4.81 | 1.12 | **1.73** | 1.34 | 5.23 | - |  |  |  |  |
| 8 | *Gracixalus sapaensis* | 4.70 | 5.19 | 2.01 | **0.93** | 1.78 | 5.69 | 2.01 | - |  |  |  |
| 9 | *Gracixalus nonggangensis* | 4.68 | 5.17 | 1.89 | **0.80** | 1.67 | 5.56 | 1.89 | 0.56 | - |  |  |
| 10 | *Gracixalus ziegleri* | 5.04 | 5.53 | 2.01 | **1.16** | 1.78 | 5.23 | 2.00 | 0.89 | 0.78 | - |  |
| 11 | *Gracixalus ananjevae* | 4.78 | 4.67 | 4.45 | **4.63** | 4.45 | 1.11 | 4.67 | 5.24 | 5.12 | 5.01 | - |
